# 478. COVID-19 maternal antibody concentrations in twin infants: impact of multiple pregnancy on transplacental antibody transfer during pregnancy

**DOI:** 10.1093/ofid/ofad500.548

**Published:** 2023-11-27

**Authors:** Alisa B Kachikis, Mindy Pike, Linda Eckert, Alexis L Baranoff, Hye Cho, Jennifer E Stolarczuk, Erin Goecker, Alexander L Greninger, Janet A Englund

**Affiliations:** University of Washington Department of Obstetrics & Gynecology, Seattle, Washington; University of Washington, Seattle, Washington; University of Washington, Seattle, Washington; University of Washington, Seattle, Washington; SUNY Upstate Medical University, Syracuse, New York; University of Washington, Seattle, Washington; University of Washington, Seattle, Washington; University of Washington, Seattle, Washington; Seattle Children’s Hospital, Seattle, Washington

## Abstract

**Background:**

COVID-19 vaccines in pregnancy protect both pregnant individuals and young infants from severe illness via transplacental transfer of maternally-derived IgG. However, the impact of multiple (e.g. twin) pregnancy on transplacental transfer of SARS-CoV-2 IgG is unknown. We aimed to evaluate anti-Spike (S) antibody transfer among infants from twin pregnancies compared to singleton pregnancies.

**Methods:**

We conducted a prospective cohort study among individuals with twin and singleton pregnancies who received at least 2 doses of an mRNA COVID-19 vaccine prior to delivery. We tested paired maternal and cord samples for anti-S IgG and used linear regression to evaluate associations between multiple or singleton pregnancy and anti-S antibody. We included as covariates timing of last vaccine dose, gestational age at delivery, number of doses prior to delivery, and small for gestational age (< 10^th^ percentile) birthweight.

**Results:**

We tested maternal/cord anti-S IgG from 25 twin and 291 singleton pregnancies. The median gestational age at delivery was 36 weeks for twin infants compared to 39 weeks for singleton infants. Median maternal anti-S IgG was 5812 BAU/mL (IQR:754, 16061) and 2971 BAU/mL (IQR:706, 14000) for twin and singleton pregnancies, respectively. Median cord anti-S IgG was 4110 BAU/mL (IQR: 527, 15937) and 3636 BAU/mL (IQR: 1019, 15465) for twin and singleton infants, respectively (Figure 1). Cord:maternal IgG ratios were significantly lower in twin pregnancies compared to singleton pregnancies (p < 0.01; Figure 2). After adjustment for covariates, there was no difference between maternal anti-S IgG concentrations (beta: -0.54; 95% confidence interval [CI]: -1.26,0.18; p=0.14), cord anti-S IgG concentrations (beta: -0.77; 95% CI: -1.68,0.13; p=0.10) or cord:maternal IgG ratios (beta: -0.07; 95% CI: -0.32,0.19; p=0.61) between twin and singleton pregnancies.

**Figure:**
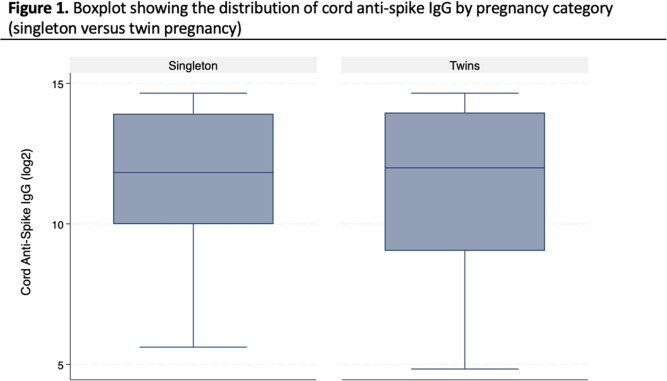

**Figure:**
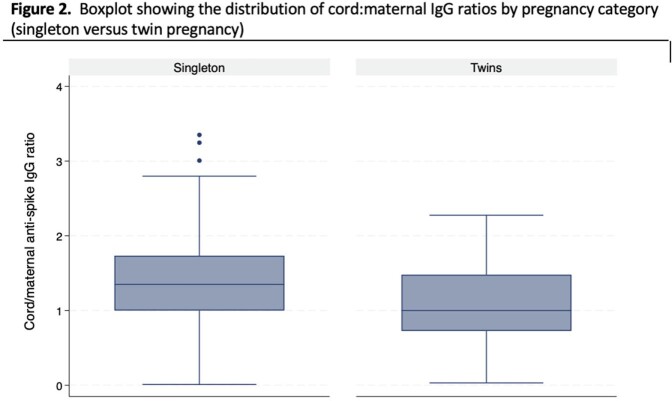

**Conclusion:**

Twin and singleton infants benefit similarly from maternal COVID-19 vaccine during pregnancy. Higher risk pregnancies including multiple pregnancies should be considered in health policy discussions regarding COVID-19 vaccine timing in pregnancy.

**Disclosures:**

**Alisa B. Kachikis, MD, MSc**, Merck: Grant/Research Support|Pfizer: Grant/Research Support **Mindy Pike, PhD**, Merck: Grant/Research Support **Alexander L. Greninger, MD, PhD**, Cepheid: central contracts|Hologic: central contracts|Janssen: central contracts|Novavax: central contracts|Pfizer: central contracts **Janet A. Englund, MD**, Ark Biopharma: Advisor/Consultant|AstraZeneca: Advisor/Consultant|AstraZeneca: Grant/Research Support|GlaxoSmithKline: Grant/Research Support|Meissa Vaccines: Advisor/Consultant|Merck: Grant/Research Support|Moderna: Advisor/Consultant|Moderna: Grant/Research Support|Pfizer: Advisor/Consultant|Pfizer: Grant/Research Support|Sanofi Pasteur: Advisor/Consultant

